# Combination secukinumab and surgical intervention for moderate-to-severe hidradenitis suppurativa: A retrospective cohort study

**DOI:** 10.1016/j.jdin.2025.08.014

**Published:** 2025-10-02

**Authors:** Qiuyun Xu, Yifan Xie, Zhuo Chen, Fang Fang, Shuyi Shen, Songyu Wang, Chengbei Bao, Ying Zou, Dan Deng, Chao Ji, Ting Gong

**Affiliations:** a Department of Dermatology, the First Affiliated Hospital of Fujian Medical University, Fuzhou, China; b Key Laboratory of Skin Cancer of Fujian Higher Education Institutions, the First Affiliated Hospital, Fujian Medical University, Fuzhou, China; c Fujian Dermatology and Venereology Research Institute, the First Affiliated Hospital, Fujian Medical University, Fuzhou, China; d Institute of Dermatology, Fujian Medical University, Fuzhou, China; e Fujian Provincial Clinical Research Center for Immune Skin Diseases, the First Affiliated Hospital, Fujian Medical University, Fuzhou, China; f Department of Dermatology, Shanghai Children’s Medical Center, School of Medicine, Shanghai Jiao Tong University, Shanghai, China

**Keywords:** hidradenitis suppurativa, HiSCR, IHS4-55, IL-17, secukinumab

*To the Editor:* Hidradenitis suppurativa (HS) is a chronic skin condition that is primarily characterized by recurrent inflammatory nodular lesions in apocrine gland–bearing skin, potentially causing permanent skin changes.^1^ Secukinumab was approved by the US Food and Drug Administration in October 2023 for the treatment of moderate-to-severe HS, establishing it as a promising therapeutic option.^2^ To date, real-world data on whether surgical intervention enhances outcomes during secukinumab treatment remain limited.

We present a retrospective analysis of 29 patients with moderate-to-severe HS who received secukinumab alone or secukinumab with surgery in the First Affiliated Hospital of Fujian Medical University. Both groups received secukinumab (300 mg once weekly for the first month and 300 mg monthly from the second month) for 48 weeks (Supplementary Methods, available via Mendeley at http://data.mendeley.com/datasets/4mtf8bwjxm/1). The surgery group additionally received a surgical intervention at baseline.

Fifteen participants were included in the surgery group, and 14 in the secukinumab monotherapy group. Two groups were comparable in age, gender, BMI, age at onset, and disease duration (Table I). The surgery group included 5 patients with Hurley stage II and 10 patients with Hurley stage III, whereas the monotherapy group had an equal number of these 2 stages. Of the surgery group, the median International Hidradenitis Suppurativa Severity Score System score at baseline was 18 (IQR: 12-23) compared to 16 (IQR: 9-31) in the monotherapy group. After 48 weeks, 5 patients achieved International Hidradenitis Suppurativa Severity Score System-55 (IHS4-55) in monotherapy, whereas 15 patients achieved it in the surgery group (Supplementary Fig 1, available via Mendeley at http://data.mendeley.com/datasets/4mtf8bwjxm/1). The surgery group showed a greater reduction in abscess and inflammatory nodule with a median of 8 (IQR: 5-11) compared to 4 (IQR: 1-5). In addition, improvement of Dermatology Life Quality Index was a greater mean in the surgery group (12.67 ± 4.78 vs 8.86 ± 2.60) (Table II). Two groups showed a significant difference in HiSCR-50, which indicated that the surgery group was more likely to have more patients achieve these 2 goals more quickly (Supplementary Fig 2, available via Mendeley at http://data.mendeley.com/datasets/4mtf8bwjxm/1). No serious adverse events were observed in the 2 groups. A detailed overview of the adverse events is shown in Supplementary Table I (available via Mendeley at http://data.mendeley.com/datasets/4mtf8bwjxm/1).

**Table I:** Patient characteristics

Characteristics	SEC + surgery (*n =* 15)	SEC monotherapy (*n =* 14)	*P* value
Age, median (IQR), y	25.00 (18.00-29.00)	22.00 (16.00-45.00)	.965
Age at onset, median (IQR), y	18 (14-30)	17 (15-19)	.677
Gender∗, *n* (%)	13 (86.7)	10 (71.4)	.580
Body mass index (kg/m^2^), mean ± SD	27.61 ± 5.01	26.34 ± 5.01	.465
Disease duration, median (IQR), y	4 (1-10)	5 (3-10)	.282
IHS4-score, median (IQR)	18 (12-23)	16 (9-31)	.570
Moderate, *n* (%)	3 (20.00)	4 (28.57)	
Severe, *n* (%)	12 (80.00)	10 (71.43)	
Hurley stage, *n* (%)			.895
Hurley stage II	5 (33.33)	7 (50.00)	
Hurley stage III	10 (66.67)	7 (50.00)	
HS-PGA, *n* (%)			.884
Moderate	3 (20.00)	3 (21.43)	
Severe	9 (60.00)	7 (50.00)	
Very severe	3 (20.00)	4 (28.57)	
Infection, *n* (%)	4 (26.67)	5 (35.71)	.700
DLQI, mean ± SD	21.00 ± 3.70	20.07 ± 4.83	.564

*DLQI*, Dermatology Life Quality Index; *HS-PGA*, Hidradenitis Suppurativa Physician’s Global Assessment Scale; *IHS4*, International Hidradenitis Suppurativa Severity Score System; *SEC*, secukinumab.

∗ Female.

**Table II:** Clinical and patient-reported outcomes after 48 weeks of treatment

Scores	SEC + surgery (*n =* 15)	SEC monotherapy (*n =* 14)	*P* value
ΔIHS4, median (IQR)	15 (9-18)	8 (5-10)	.001∗
IHS4-55, *n* (%)	15 (100.0)	5 (35.7)	<.001∗
≥2 change in HS-PGA, *n* (%)	15 (100.0)	12 (85.7)	.224
HiSCR-50, *n* (%)	15 (100.0)	10 (71.4)	.042∗
ΔAN-count, median (IQR)	8 (5-11)	4 (1-5)	.001∗
ΔDLQI, mean ± SD	12.67 ± 4.78	8.86 ± 2.60	.023∗

*ΔAN-count*, Difference in change of abscess and inflammatory nodule count; *ΔDLQI*, difference in change of Dermatology Life Quality Index; *HiSCR*, Hidradenitis Suppurativa Clinical Response; *HS-PGA*, Hidradenitis Suppurativa Physician’s Global Assessment Scale; *IHS4*, International Hidradenitis Suppurativa Severity Score System; *ΔIHS4*, difference in International Hidradenitis Suppurativa Severity Score; *SEC*, secukinumab.

∗ Significant values.

Biologic agents have emerged as an increasingly used therapeutic approach for HS. Meanwhile, deroofing, limited, or wide excisions are frequently used as additional surgical therapy in HS clinical practice.^3^ When combined with biologic and other medical treatments, surgical procedures effectively remove chronic, deep, confluent, interconnected epithelialized tunnels exhibiting bacterial overgrowth and biofilm formation. Surgical interventions reduce the overall inflammatory burden, enhance the effectiveness of concomitant biologic therapy, and increase the likelihood of achieving remission. Studies suggest that not only is it likely safe to continue biologics during surgery in patients with HS undergoing surgery, but patients maintained on biologics also appear to experience better surgical outcomes.^3^^,^^4^

Our research suggested that secukinumab combined with surgery resulted in significant alleviation of disease and enhanced quality of life in patients with moderate-to-severe HS. Despite small sample size limitations and a lack of a surgery-only group in our research, it should be recommended that surgery combined with biologics could be a treatment option for patients with moderate-to-severe HS.

### Declaration of generative AI and AI-assisted technologies in the writing process

During the preparation of this work the authors used ChatGPT in order to improve language and readability. After using this tool/service, the authors reviewed and edited the content as needed and take full responsibility for the content of the publication.

## Conflicts of interest

None disclosed.
